# Copper-Based Nanopesticides at the Benthic Interface: Transformation, Speciation, Invertebrate Exposure, and Food-Web Risks

**DOI:** 10.3390/toxics14080681

**Published:** 2026-08-03

**Authors:** Xin Wu, Yuling Dong, Ao Li, Changjian Xie

**Affiliations:** 1 School of Life Sciences and Medicine, Shandong University of Technology, Zibo 255000, China; wuxin010216@163.com (X.W.); yuling-dong@163.com (Y.D.); acqx@163.com (A.L.); 2 School of Geography, Earth & Environmental Sciences, University of Birmingham, Edgbaston, Birmingham B15 2TT, UK

**Keywords:** copper-based nanopesticides, CuO nanoparticles, benthic interface, synchrotron speciation, lower-trophic invertebrates, trophic transfer, food-web risk, bioavailability, ecological risk assessment

## Abstract

Copper-based nanopesticides are increasingly explored as nano-enabled alternatives to conventional copper pesticides because they may improve deposition, antimicrobial efficacy, and material-use efficiency. Their environmental risk, however, cannot be inferred from total copper concentration or from the toxicity of pristine particles alone. After agricultural application, copper-based nanoforms may be transported to soils, drainage waters, wetlands, and sediments, where aggregation, dissolution, aging, sulfidation, organic complexation, and biological processing reshape their speciation and bioavailability. This review critically examines copper-based nanopesticides at the benthic interface, with emphasis on environmental transformation, synchrotron-resolved speciation, lower-trophic invertebrate exposure, trophic transfer, and food-web risk. We highlight that sediment-associated organisms are not only toxicity endpoints but also biological processors and vectors of transformed copper species. Evidence from stable-isotope tracing, dietary exposure studies, mesocosms, and micro-food-web experiments shows that copper-based nanoforms can enter aquatic, benthic, and terrestrial food chains. However, most studies demonstrate transfer or accumulation rather than consistent biomagnification across trophic levels. We further argue that future risk assessment should move beyond single-material and single-endpoint testing toward transformation-aware, route-specific, and food-web-relevant frameworks. Integrating total copper analysis with particle-specific measurements, synchrotron-based speciation where analytically feasible, realistic lower-trophic exposure models, and ecosystem-level endpoints will be essential for evaluating the long-term risks of copper-based nanopesticides.

## 1. Introduction

Copper-based pesticides have been used for more than a century to suppress fungal and bacterial diseases in agriculture. Conventional products, including copper sulfate, Bordeaux mixture, copper oxychloride, and copper hydroxide, remain important because they provide broad-spectrum antimicrobial activity and a relatively low risk of resistance development. At the same time, repeated copper application can increase copper loading in agricultural soils and receiving environments, creating a long-standing tension between crop protection and environmental accumulation. Nano-enabled copper formulations have been developed to improve foliar deposition, persistence, antimicrobial efficacy, and material-use efficiency while reducing active-ingredient inputs [1,2]. These formulations belong to a broader class of nanopesticides whose environmental fate, exposure pathways, and risk profiles may differ from those of conventional pesticide products [3,4]. For copper-based nanopesticides, these benefits and uncertainties converge around the same issue: the nanoscale form may improve crop protection, but it may also alter dissolution kinetics, particle persistence, surface reactivity, biological uptake, and non-target interactions [5].

**Figure 1:**
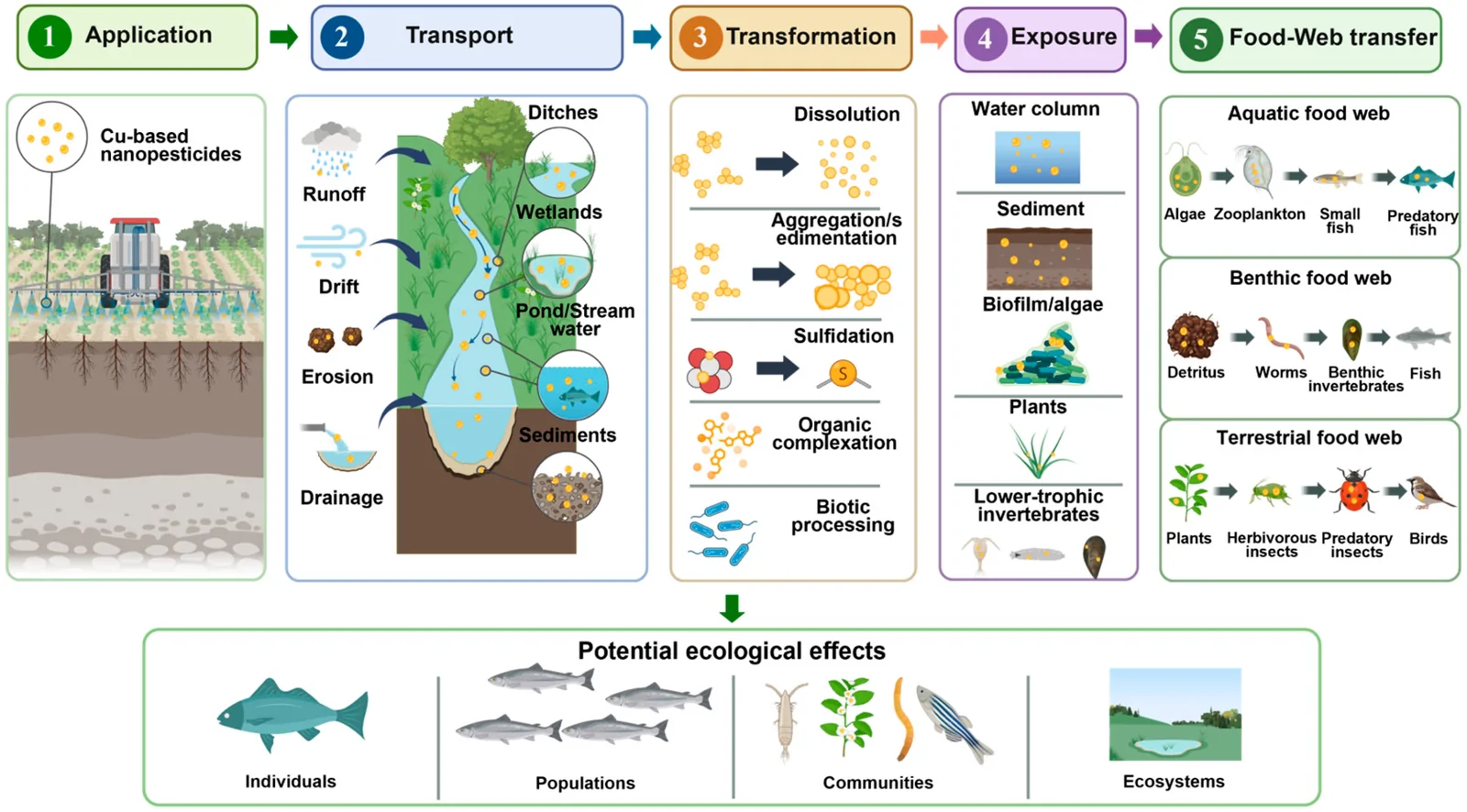
Conceptual framework linking copper-based nanopesticide inputs with environmental transport, benthic-interface transformation, lower-trophic exposure, and food-web risk. Copper-based nanoforms released from agricultural systems may be transported to receiving waters and sediments, where aggregation, dissolution, aging, sulfidation, organic complexation, sediment binding, and remobilization reshape copper speciation and bioavailability. Lower-trophic organisms can acquire transformed copper through sediment ingestion, biofilm feeding, algal grazing, or predation, thereby linking benthic transformation with trophic transfer and ecosystem-level consequences. Figure created with Biorender.com.

These uncertainties become more important once copper-based nanopesticides leave the target crop surface. Through runoff, drainage, spray drift, or soil erosion, CuO, Cu(OH)_2_, and related copper nanoforms may enter soils, ditches, ponds, wetlands, streams, and freshwater sediments. In these receiving systems, they can aggregate, sediment, dissolve, and complex with natural organic matter [6]. They may also interact with mineral surfaces or transform into reduced and sulfur-associated copper phases [7,8]. As a result, ecological exposure is not governed simply by the applied dose or total copper concentration. It is governed by how copper moves among particulate, dissolved, organic-complexed, mineral-bound, and biologically transformed forms. This transformation perspective makes the benthic interface a particularly important risk hotspot. Sediments can retain copper-bearing particles and colloids, but they are not passive sinks. Redox gradients, sulfide production, microbial activity, organic matter turnover, porewater chemistry, pH heterogeneity, and particle resuspension can immobilize copper, convert it into new species, or remobilize it into bioavailable forms. These processes are especially relevant for nanopesticides because aged and sediment-associated copper forms may differ substantially from freshly dispersed particles used in standard laboratory tests. Figure 1 summarizes this review framework, in which agricultural inputs are linked to benthic transformation, copper speciation, lower-trophic invertebrate exposure, and food-web consequences.

Understanding copper-based nanopesticides at this interface requires more than bulk elemental analysis. ICP-MS can quantify total copper with high sensitivity, but it cannot distinguish residual CuO, dissolved Cu, Cu–organic complexes, Cu–sulfide phases, or mineral-associated copper. This distinction matters because these forms differ in solubility, mobility, gut lability, biological uptake, and toxicity. Synchrotron-based approaches provide complementary information: X-ray absorption near-edge structure (XANES), extended X-ray absorption fine structure (EXAFS) constrain Cu oxidation state and local coordination, whereas micro-X-ray fluorescence (μXRF) maps elemental Cu distribution in complex environmental and biological matrices [9]. Together, these methods provide a mechanistic bridge between environmental chemistry and ecotoxicology, although they do not independently confirm intact nanoparticle identity.

Lower-trophic invertebrates provide the biological link between benthic transformation and food-web exposure. By feeding on sediments, biofilms, algae, detritus, or contaminated prey, they can acquire transformed copper species and redistribute them across environmental and biological compartments. They should therefore be viewed not only as toxicity endpoints but also as biological processors and vectors of copper-based nanoforms. Evidence from sediment-based, aquatic, and terrestrial food-chain studies indicates that copper-based nanoforms can move across trophic levels, although available data support context-dependent transfer rather than universal biomagnification [10,11,12]. This distinction is important because transfer depends on copper transformation, prey processing, gut chemistry, and predator assimilation.

The remaining knowledge gap is not simply a lack of toxicity data. The key limitation is that copper chemistry and ecological function are often studied separately, making it difficult to determine which transformed copper forms actually drive exposure, toxicity, and food-web propagation. Many studies still report total copper accumulation or apical endpoints such as mortality, growth inhibition, reproduction, and oxidative stress, while fewer connect copper speciation with exposure route, biological processing, trophic movement, and ecosystem function. This separation is particularly problematic for copper-based nanopesticides because the same total copper burden may reflect residual particles, dissolved ions, sulfur-associated phases, organic complexes, sediment-bound copper, or biologically transformed species. Recent mesocosm and micro-food-web studies suggest that ecological risk may emerge through altered community structure, trophic interactions, and nutrient cycling, not only through direct toxicity to individual organisms [13,14].

Here, we examine copper-based nanopesticides through the lens of the benthic interface, where environmental transformation, copper speciation, lower-trophic exposure, and food-web consequences intersect. By connecting copper chemistry with ecological function, we aim to clarify when copper-based nanoforms remain localized, become bioavailable, or propagate through ecological networks.

## 2. Materials and Methods

This review was developed as a critical narrative review with a scoping component, rather than as a quantitative systematic review or meta-analysis. This approach was intended to provide a transparent literature scope rather than an exhaustive systematic search. The literature was identified from major scientific databases, including Web of Science, Scopus, PubMed, ScienceDirect, and Google Scholar. The search focused primarily on studies addressing copper-based nanopesticides, CuO NPs, copper hydroxide nanoformulations, environmental transformation, copper speciation, benthic exposure, lower-trophic organisms, trophic transfer, food-web effects, and ecological risk assessment. Earlier studies were also included when they provided essential mechanistic evidence on copper dissolution, sediment aging, dietary exposure, synchrotron-based speciation, or nanoparticle trophic transfer. The literature scope included original research articles and relevant review papers. Original studies were prioritized when they provided experimental evidence on copper transformation, speciation, bioavailability, organismal uptake, toxicity, trophic transfer, or community-level effects. Review papers were used mainly to establish broader context, identify conceptual gaps, and support the interpretation of evidence across environmental chemistry and ecotoxicology. Because nanopesticides represent a broad formulation category rather than a single material type, studies were interpreted according to copper chemistry, transformation state, exposure route, biological model, and ecological context [15,16,17].

The review considered nano-enabled copper hydroxide formulations, CuO NPs, commercial copper-based nanopesticides, and environmentally transformed copper phases relevant to agricultural runoff and sedimentary systems. These transformed phases included Cu_2_O, CuS/Cu_x_S, Cu–organic complexes, and mineral-associated copper species. Studies on non-pesticidal CuO NPs and copper salts were included when they provided mechanistic insight into dissolution, aging, speciation, bioavailability, dietary exposure, copper detoxification, or food-chain transfer. Studies focused solely on material synthesis, antimicrobial coatings, biomedical applications, catalytic activity, or mammalian toxicology were excluded unless they provided information directly relevant to copper speciation or environmental transformation. The synthesis was structured to connect copper transformation and speciation with lower-trophic exposure, trophic transfer, food-web effects, and risk mitigation. Particular attention was given to studies that linked copper chemistry with biological uptake, organism-level responses, community-level effects, or risk interpretation.

The biological scope focused on lower-trophic organisms that link particle-associated contamination with food-web exposure, including oligochaetes, chironomid larvae, nematodes, daphnids, brine shrimp, mollusks, earthworms, and planarians. Vertebrate studies were considered only when they were part of food-chain designs or helped interpret transfer from lower-trophic organisms to higher consumers. Plant studies were included when they addressed copper-based nanopesticide uptake, transformation, or transfer to herbivores.

## 3. Environmental Transformation at the Benthic Interface

The environmental behavior of copper-based nanopesticides is best understood as a transformation continuum rather than a single fate pathway. After agricultural application, CuO NPs, Cu(OH)_2_ nanopesticides, and commercial copper-based formulations may be transported from crop surfaces and soils into drainage systems, ponds, wetlands, and streams. Mesocosm and soil-solution studies show that CuO and Cu(OH)_2_ nanoforms can persist as particles while partial dissolution proceeds [18,19]. Natural waters can further alter copper-based nanoforms through aggregation, sedimentation, dissolution, and secondary transformation. The direction and rate of these processes depend on ionic strength, pH, phosphate, and dissolved organic carbon [20]. For benthic environments, this means that incoming copper is rarely present as only pristine particles or only dissolved ions. It is more often a mixture of particle-bound, dissolved, colloidal, complexed, and sediment-associated copper forms.

Aggregation and sedimentation are usually the first processes shifting copper-based nanoforms from the water column to the benthic compartment. Electrolytes can compress particle surface charge, while divalent cations, phosphate, clay minerals, and natural organic matter may either accelerate heteroaggregation or stabilize particles through surface coating. For Cu(OH)_2_ nanorod-based nanopesticides, aggregation, sedimentation, and dissolution varied markedly among soil solutions, indicating that soil pH, organic matter, and texture can regulate nanopesticide stability before particles reach receiving waters [19]. In natural waters, copper nanoparticles also show medium-dependent sedimentation and transformation, which means that mobility cannot be inferred from ultrapure water tests alone [20]. These processes reduce water-column residence time but may increase sediment accumulation and benthic exposure.

Dissolution controls the formation of labile copper species, but it should not be interpreted as rapid and complete conversion to free Cu^2+^. In freshwater mesocosms, Kocide 3000, a commercial copper hydroxide pesticide containing nanoscale Cu(OH)_2_ particles, dissolved faster than CuO NPs, yet both materials persisted as particles over environmentally relevant timescales [18]. Soil studies further show that pH and organic matter regulate the solubility and dissolution rate of CuO NPs in different ways [21]. Dissolved organic matter, plant exudates, bacteria, and particle coatings can also control CuO NPs dissolution at alkaline pH [22]. These findings are important for benthic risk because slowly dissolving particles may settle before dissolution is complete, creating mixed exposure to residual particles, dissolved copper, and ligand-bound copper.

Redox and ligand-driven transformations add further complexity at the sediment–water interface. Sediments often contain microsites with low oxygen, high microbial activity, and elevated reduced sulfur species. Under such conditions, CuO NPs can undergo sulfidation, with X-ray absorption spectroscopy showing transformation from crystalline CuO to amorphous Cu_x_S and then to more crystalline CuS-like phases [8]. The oxic–anoxic boundary commonly occurs within the upper millimeters of sediment and shifts with oxygen demand, organic-matter loading, hydrodynamic disturbance, and bioturbation [23]. Copper-bearing particles can therefore move between oxic, suboxic, and sulfidic microzones over relatively short vertical distances. Formation of poorly ordered CuxS and its subsequent development into more crystalline CuS-like phases depends on sulfide supply, particle residence time, and local redox conditions [8]. Increased structural order may alter reaction kinetics, but crystallinity alone does not establish permanent immobilization because sediment mixing and reoxygenation can expose both poorly ordered and crystalline phases to renewed transformation. Sulfidation can reduce copper solubility and short-term bioavailability, but it should not be treated as permanent detoxification. Sulfur-associated copper may be reoxidized, physically resuspended, or ingested by benthic organisms. Organic ligands create another pathway. Humic substances, root exudates, extracellular polymeric substances, and microbial metabolites can stabilize dissolved or colloidal copper, while thiol-containing ligands may promote Cu binding and partial reduction [22]. Under reducing and sulfide-rich conditions, Cu(I) can be stabilized through Cu–S coordination, whereas uncomplexed aqueous Cu(I) is not expected to persist in oxic waters [24]. Thus, the same transformation that decreases free Cu^2+^ may still maintain copper in mobile, colloidal, or biologically accessible forms. Gut passage creates a chemical environment that can differ markedly from the surrounding porewater. Changes in pH, ionic strength, redox state, digestive ligands, amino acids, and microbial activity may dissolve or restructure ingested Cu phases. In the isopod *Porcellio scaber*, most Cu accumulated after Cu NP exposure was attributed to ions released within the digestive tract [25]. Although direct data for benthic invertebrates remain limited, ex vivo fish gut studies provide complementary evidence that low pH and amino acids can increase the dissolution and bioavailability of CuO nanomaterials [26]. A Cu phase that is sparingly soluble in sediment may therefore remain gut-labile, while dissolution within the gut does not necessarily imply assimilation because Cu may still be rebound or egested.

Aging should be interpreted as redistribution among copper pools rather than disappearance of copper risk. In five soils, short-term reactions of dissolved Cu, CuO NP, and CuS NP depended on both initial copper form and soil chemistry, whereas longer-term speciation became increasingly controlled by soil properties [27]. This finding helps explain why freshly prepared exposure systems may overestimate nano-specific behavior but underestimate long-term sediment retention. In sediment-dwelling oligochaetes, stable-isotope experiments showed that aging of spiked sediment reduced the body burden of both CuO NP-derived copper and dissolved copper in *Tubifex tubifex*, but exposure was not eliminated [28]. This distinction is critical because porewater chemistry alone may underestimate dietary uptake of aged or sediment-associated copper.

The benthic interface also creates feedbacks between biological activity and copper transformation. Microbial biofilms can release extracellular polymeric substances, organic acids, thiols, and redox-active metabolites that modify copper dissolution and complexation. Burrowing and bioturbation by oligochaetes, chironomid larvae, and other sediment-dwelling organisms can redistribute particles, alter oxygen penetration, and expose reduced copper phases to oxidizing conditions. Plant roots and rhizosphere microorganisms may further alter copper speciation through ligand release, pH modification, and redox reactions. In the soil–rice system, CuO NPs were transformed during the rice life cycle, showing that plant-associated environments can actively reshape copper fate rather than simply receive copper passively [29].

These transformation pathways create the analytical problem addressed in the next section. Total copper measurements are necessary for mass balance, but they cannot identify the species that control mobility, bioavailability, dietary uptake, or toxicity. Table 1 summarizes the major transformation processes expected at the benthic interface and indicates how each process may shift copper exposure from the water column to sediments, porewaters, lower-trophic organisms, and food webs.

## 4. Synchrotron-Resolved Copper Speciation and Spatial Mapping

**Figure 2:**
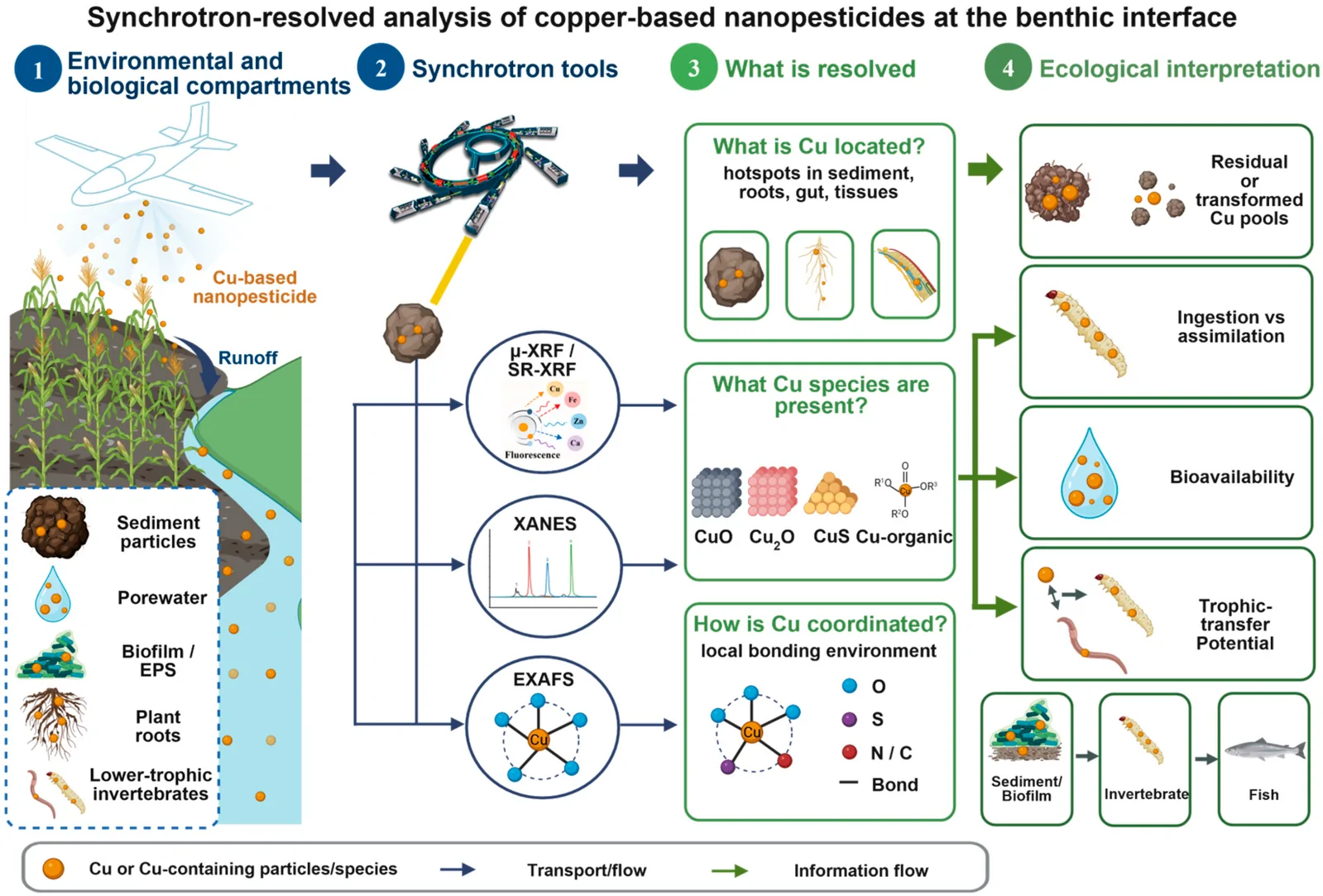
Conceptual illustration of synchrotron applications in copper-based nanopesticide research. Environmental and biological samples from the benthic interface, including sediments, porewater-associated fractions, biofilms, plant roots, and lower-trophic organisms, can be analyzed using μ-XRF/SR-XRF, XANES, and EXAFS to determine copper localization, oxidation state, and coordination environment. Integrating these outputs constrains Cu localization and local chemical environment, but confirmation of intact nanoparticles requires complementary particle-resolved evidence. Figure created with Biorender.com.

The transformation pathways summarized in Table 1 create a central analytical challenge for copper-based nanopesticide research. Total copper measurements can quantify accumulation and support mass balance, but they cannot distinguish residual CuO, Cu(OH)_2_, Cu_2_O, CuS/Cu_x_S, Cu–organic complexes, mineral-associated copper, or dissolved ionic species. This distinction is essential because these forms differ in solubility, persistence, gut lability, biological uptake, and toxicity. Synchrotron-based methods are therefore most useful when they are used to answer a specific mechanistic question: which copper species are present, where they are located, and how their distribution changes across environmental and biological compartments (Figure 2).

X-ray absorption spectroscopy provides chemically specific evidence on Cu oxidation state and local coordination in complex environmental and biological matrices [30,31]. Rather than serving as a general characterization tool, XANES and EXAFS are most informative when used to separate environmentally meaningful Cu pools. XANES is sensitive to oxidation state and near-edge coordination, whereas EXAFS provides information on neighboring atoms, bond distances, and local structural order. These measurements can distinguish sufficiently different Cu environments, such as Cu–O- and Cu–S-dominated coordination, but they do not directly reveal particle morphology or origin [31,32]. A CuO-like spectral contribution should therefore be interpreted as a CuO-like local environment rather than proof that the originally applied nanoparticle remains intact. This distinction is particularly important in soils and sediments, where Cu can redistribute among minerals, organic matter, porewater, and biological surfaces during aging and redox cycling.

Spatial mapping adds a second layer of interpretation that bulk spectroscopy cannot provide. μ-XRF and related synchrotron-based mapping tools can show where copper is concentrated within heterogeneous matrices, including biosolids, sediments, roots, and biological tissues [33]. Synchrotron-based XRF imaging in plants also provides a useful methodological reference for future copper nanopesticide studies in lower-trophic organisms [34]. For benthic exposure, this spatial dimension is essential because copper located in gut contents, epithelial surfaces, storage granules, or excretory structures has different implications for ingestion, assimilation, detoxification, toxicity, and trophic transfer. However, a Cu hotspot identified by μ-XRF represents elemental enrichment rather than a specific Cu phase, and its interpretation requires XAS or complementary particle-resolved analysis [31].

Several environmental and plant studies show how synchrotron approaches can move copper nanopesticide research beyond bulk accumulation. In soils, XRF and XANES showed that nanosized CuO underwent size-dependent dissolution followed by adsorption to soil materials, while microbial responses were shaped more strongly by land-use history than by copper fate alone [9]. In CuO NP-amended soil, XANES indicated that aging time and nanoparticle concentration altered Cu extractability by changing the relative contributions of CuO-like and secondary Cu coordination environments [35]. These findings show why total Cu alone is insufficient for interpreting exposure. Similar total concentrations may reflect different proportions of CuO-like, adsorbed, and transformed Cu environments, although XANES cannot independently determine whether the CuO-like fraction remains as intact primary particles.

Freshwater wetland mesocosms provide a more direct environmental bridge to benthic systems. X-ray-based speciation indicated rapid transformation of CuO NPs in surficial sediment, whereas a CuO-like coordination environment persisted longer in aquatic plant tissues [36]. This contrast is important because it shows that transformation rates measured in sediment cannot be assumed to represent transformation in biota. It also suggests that exposure at the benthic interface may involve both rapidly transformed sediment-associated copper and more persistent biologically retained copper forms.

Plant-associated systems provide additional evidence that biological compartments can actively reshape copper species. In rice plants exposed to CuO NPs, synchrotron-based analyses showed copper translocation and in vivo biotransformation [37]. In wheat roots exposed to CuO, Cu(OH)_2_, and CuS NPs, temporal mapping revealed that the initial copper form influenced both localization and speciation over time [38]. These studies are not direct lower-trophic invertebrate experiments, but they are useful analogs. They demonstrate that biological surfaces, internal ligands, tissue architecture, and exposure time can determine whether copper remains particle-like, dissolves, or becomes associated with new biological binding environments. These studies resolve changes in Cu localization and local coordination, but persistence of intact nanoparticles cannot be concluded without complementary imaging, diffraction, or particle-specific measurements [31,37,38].

For lower-trophic invertebrates, a major analytical objective is to distinguish ingestion from assimilation. Whole-body ICP-MS measures Cu associated with the recovered organism, but external adsorption and gut contents may contribute unless surface cleaning, depuration, dissection, or tissue-specific analysis is performed. μ-XRF or SR-XRF can localize Cu-rich regions, although elemental mapping alone does not demonstrate tissue assimilation [31]. When Cu hotspots are selected for XANES or EXAFS, spatial and chemical information can be combined within the same specimen. This approach would be particularly valuable for oligochaetes, chironomid larvae, mollusks, and planarians, where sediment ingestion, gut transformation, tissue uptake, and trophic transfer may occur together.

The interpretation of synchrotron data requires caution. Drying, chemical fixation, sectioning, and beam exposure may alter Cu distribution or redox-sensitive species, particularly in hydrated sediments, biofilms, and soft tissues [32]. Cryogenic fixation, transfer, and measurement can better preserve hydration and elemental localization, although they do not eliminate beam-induced changes and should be accompanied by dose-dependent checks [39]. Spectral interpretation also depends on the environmental relevance and completeness of the reference library. Residual CuO and secondary Cu-O or Cu-S phases may have overlapping local structures, making linear-combination fitting non-unique and minor components difficult to quantify [31,32]. Analytical sensitivity presents an additional constraint. Fluorescence EXAFS has been obtained from solutions containing 1.2–4.4 ppm Cu [24], but low-ppb porewaters are generally below the practical range of bulk XAS without enrichment. Targeting μ-XRF hotspots can improve the signal, but the resulting spectra represent Cu-enriched regions rather than the average environmental compartment. Synchrotron results should therefore be interpreted with mass balance, particle-specific measurements, microscopy, isotope tracing, and relevant environmental chemistry (Table 2).

In the context of this review, synchrotron-resolved speciation should be treated as a mechanistic bridge rather than a standalone characterization step. Its strongest contribution arises when spatial and chemical information is integrated with mass balance, particle characterization, and biological processing. The most informative designs would compare pristine and aged copper-based nanopesticides, sediment-bound and porewater-associated fractions, gut-content and tissue-associated copper, and prey-to-predator transfer. Such comparisons can help determine whether benthic exposure is driven mainly by residual particles, transformed sediment-bound species, labile dissolved copper, or biologically processed copper. This distinction is necessary before lower-trophic invertebrate toxicity and trophic-transfer data can be interpreted mechanistically.

## 5. Lower-Trophic Invertebrate Exposure and Toxicity

Lower-trophic organisms are the first biological interface between transformed copper species and food-web transfer. In agricultural ditches, ponds, wetlands, soils, and sediments, they rarely encounter copper-based nanopesticides as freshly dispersed particles in clean water. Instead, exposure usually involves mixtures of dissolved, colloidal, sediment-bound, food-associated, and aged copper species. These forms can enter organisms through waterborne, dietary, sediment-associated, or biofilm-mediated routes [40,41]. Lower-trophic toxicity should therefore be interpreted in relation to copper speciation, exposure route, and feeding ecology rather than total copper concentration alone.

Freshwater invertebrate studies show that exposure route can strongly alter copper nanoparticle uptake and toxicity. In *Daphnia magna*, CuO NPs can be ingested during acute exposure and are largely associated with the digestive tract, indicating that particle uptake is closely linked to feeding and gut processing [42]. Chronic comparisons between CuO NPs and copper salts further show that particulate and ionic copper can produce different accumulation and toxicity patterns [43]. Route-comparison studies have shown that CuO NP uptake and toxicity in *Daphnia magna* differ between algae-mediated dietary exposure and direct waterborne exposure [40]. Similar route-dependent responses have been reported in freshwater snails after waterborne and diet-borne exposure to CuO NPs [41]. These findings indicate that dietary exposure is not a secondary pathway for grazing and particle-feeding organisms.

Food availability and test-medium chemistry further modify these responses. In freshwater microcrustaceans, algae and exposure media can change nanoparticle aggregation, dissolution, ingestion, and apparent toxicity [44]. This is highly relevant for copper-based nanopesticides because runoff waters and benthic habitats contain natural colloids, dissolved organic matter, microbial exudates, algae, and detritus. Food particles may reduce toxicity by binding labile copper, but they may also increase exposure by acting as carriers of particle-bound copper. The direction of the response depends on the balance among reduced dissolved copper, increased ingestion, gut-mediated dissolution, and elimination.

Sediment-dwelling organisms are especially relevant to benthic-interface risk. Oligochaetes such as *Tubifex tubifex* continuously ingest sediment and can accumulate copper derived from both CuO NPs and dissolved copper in spiked natural sediments [10]. Long-term aging generally reduces copper body burdens in *Tubifex*, but it does not eliminate exposure when animals ingest sediment particles directly [28].

Biodynamic models use concentration–time data from uptake and depuration phases to estimate uptake, dietary assimilation, and elimination rates. Ramskov et al. [45] used this approach to separate waterborne and sediment-associated Cu exposure in *Tubifex tubifex* and to estimate the contribution of ingested Cu. Toxicokinetic analysis has similarly been applied to distinguish accumulation and elimination patterns in the deposit-feeding snail *Potamopyrgus antipodarum* [46]. By contrast, a BAF is a net organism-to-water concentration ratio that integrates several uptake and loss processes without identifying their individual contributions [47]. Biodynamic models are therefore more informative for route-specific mechanisms, although they require time-series data and complement rather than replace BAFs used for regulatory comparison.

Deposit-feeding mollusks provide additional evidence that sediment-associated copper form matters. In *Potamopyrgus antipodarum*, sediments spiked with aqueous Cu, nano-CuO, or micro-CuO produced different bioaccumulation, toxicokinetic, and effect profiles [46]. The shape of CuO NPs also altered bioavailability and toxicity in the same species, showing that sediment-associated exposure depends on particle properties as well as copper dose [48]. Whole-sediment tests with the bivalve *Scrobicularia plana* further showed that CuO NPs and soluble copper can trigger distinct metal-handling strategies, including differences in subcellular distribution and detoxification responses [49]. These studies broaden the evidence base beyond pelagic test organisms and support the inclusion of deposit-feeding and sediment-associated species in copper nanopesticide assessment.

Soil-dwelling invertebrates provide complementary evidence that copper nanoform toxicity depends on life stage, exposure duration, and biological organization. In *Enchytraeus crypticus*, CuO nanomaterial effects were life-stage dependent, and full life-cycle testing revealed responses that could be missed by standard reproduction assays [50]. Metabolomic analysis in the same species showed that CuO nanomaterials and CuCl_2_ can trigger different biochemical response patterns, including changes in amino acids, lysophospholipids, energy metabolism, neurotransmission, and immune defense [51]. Full life-cycle testing with *Eisenia fetida* also showed that earthworm responses to CuO nanomaterials can vary across developmental stages [52]. These studies move the discussion beyond acute lethality and show that copper nanoforms may affect growth trajectories, developmental timing, metabolism, and reproductive output.

Mechanistically, copper-based NPs can affect lower-trophic organisms through both dissolved and particle-associated pathways. Dissolved copper mainly acts through labile copper pools that interfere with cellular homeostasis and redox regulation, whereas particle-associated copper can accumulate in the gut, interact with digestive fluids, and generate localized exposure before dissolution or elimination. This distinction is important because gut-retained particles may alter feeding, digestion, assimilation, and internal copper handling without producing the same response pattern as dissolved copper. In *Caenorhabditis elegans* (*C. elegans*), CuO NPs affected multiple toxicological endpoints and caused neurodegeneration, supporting the inclusion of neurobehavioral endpoints in lower-trophic risk assessment [53]. Parental exposure to CuO NPs also caused transgenerational developmental and reproductive toxicity in *C. elegans*, with possible involvement of epigenetic regulation [54]. These findings indicate that short-term survival and reproduction assays may underestimate delayed, inherited, or behaviorally mediated consequences of sublethal exposure.

The gut microbiota–host axis provides another mechanism linking lower-trophic exposure with ecological function. In *Eisenia fetida*, in vivo exposure to CuO NPs shifted the gut microbiome and was examined in relation to host immune responses and susceptibility to bacterial challenge [55]. In *Enchytraeus crypticus*, CuO NPs altered gut microbial communities and resistome profiles, even though ionic copper produced stronger bioaccumulation and some stronger organism-level effects [56]. These microbial responses are ecologically relevant because gut-associated microbes contribute to digestion, nutrient processing, defense, detoxification, and gut-mediated transformation of ingested particles.

Lower-trophic invertebrate toxicity therefore cannot be reduced to a binary comparison between particles and ions. The relevant exposure unit may change across water, sediment, food, biofilm, gut contents, and internal tissues. This complexity is why lower-trophic organisms are central to copper nanopesticide risk. They integrate transformed exposure from sediments and food, generate biological responses at multiple levels, and provide a pathway through which copper can move from benthic compartments into food webs.

## 6. Trophic Transfer and Food-Web Consequences

Trophic transfer is a key pathway by which copper-based nanopesticides may move beyond organisms directly exposed at the sediment–water or soil–plant interface. For dissolved copper, food-chain transfer is usually interpreted through uptake, assimilation, detoxification, and excretion. For copper-based nanoforms, the process is more complex because copper can move as dissolved ions, residual particles, transformed particles, prey-associated residues, gut-labile species, or tissue-assimilated copper. Transfer should therefore be distinguished from biomagnification. Transfer indicates movement from prey to predator, whereas biomagnification requires a higher concentration in the consumer than in its diet or prey on a directly comparable basis. Current evidence supports context-dependent transfer of copper-based nanoforms, but not a general assumption of universal biomagnification [57,58]. The quantitative terms used for Cu accumulation and food-chain movement are not interchangeable. BCF is the ratio of organism concentration to water concentration when uptake occurs from water alone, whereas BAF uses the same water-referenced ratio but includes uptake from water, food, sediment, and other environmental routes [47,57]. BMF is calculated as the concentration in a consumer divided by that in its diet or prey, using comparable tissues and the same wet- or dry-weight basis. TMF is derived across multiple trophic levels as 10^β^, where β is the slope of log_10_-transformed concentration against trophic position [47]. The term TTF is less standardized and must be defined from the equation used in each study. A TTF above one supports biomagnification only when the numerator, denominator, tissue basis, depuration procedure, and normalization are directly comparable. Detection of Cu or an isotope tracer in a predator establishes transfer, but not necessarily tissue assimilation or biomagnification [10,59].

The most relevant evidence for benthic food-chain transfer comes from stable-isotope tracing and sediment-based exposure designs. Using ^65^Cu-enriched CuO NPs and ^65^CuCl_2_, Lammel et al. showed that newly added copper could be distinguished from background copper in natural sediment. The sediment-dwelling oligochaete *Tubifex tubifex* accumulated ^65^Cu from both nanoparticle and dissolved copper treatments, and contaminated worms subsequently transferred ^65^Cu to the fish *Gasterosteus aculeatus* [10]. A related proof-of-concept study using spiked sediments and worm-homogenate food packages further showed that CuO NPs and dissolved Cu can move through a sediment–worm–fish exposure design, while a large fraction of particulate Cu may be egested rather than assimilated by fish [59]. These studies demonstrate accumulation in worms and prey-to-predator transfer of newly introduced Cu. They do not independently establish biomagnification because directly comparable predator-to-prey concentration ratios were not the primary endpoint and a substantial fraction of ingested particulate Cu could be egested [10,59]. Aging can further reduce the bioavailability of sediment-associated copper, although it does not eliminate exposure through sediment ingestion [28]. These findings show that the benthic route is real but strongly controlled by sediment binding, aging, prey burden, gut retention, egestion, and predator assimilation.

Aquatic grazing food chains provide complementary evidence. In a simulated *Pseudokirchneriella subcapitata–Daphnia magna–Limnomysis benedeni* food chain, the mass-based TTF for particulate Cu was above one from the alga to the mysid, which the authors interpreted as biomagnification [60]. This result is specific to the particulate-Cu metric and the tested food-chain design and should not be generalized to total Cu or other trophic systems. The same study showed that particle-number-based uptake was dominated by the dietary route, illustrating that the interpretation of trophic transfer can depend on the selected mass- or particle-based metric [60]. Route-specific studies in *Daphnia magna* further show that algae-mediated dietary exposure and direct waterborne exposure can produce different CuO NP uptake and toxicity profiles [40]. Together, these findings identify algal and biofilm pathways as potential entry routes while showing that transfer efficiency depends on prey processing, consumer physiology, and the metric used.

Diet-borne exposure is also relevant for fish and other higher consumers. Feeding fish larvae with CuO NP-exposed *Artemia salina* increased whole-body Cu and demonstrated prey-mediated transfer [11]. Because the measurement was based on whole-body Cu, it does not by itself distinguish gut-associated Cu from tissue-assimilated Cu or establish biomagnification. In goldfish, waterborne exposure to CuO NPs produced stronger accumulation than dietary exposure, although contaminated prey still represented a potential transport pathway [61]. Dietary exposure studies in rainbow trout further showed that CuO engineered nanomaterials and copper sulfate can both elevate tissue copper, with the intestine and liver acting as important accumulation or handling compartments [62]. These studies support a cautious interpretation: dietary transfer may be lower than waterborne accumulation in some fish systems, but it remains relevant for food-web movement and internal copper handling (Table 3).

Terrestrial evidence is more limited but particularly important for the nanopesticide context. The clearest example involves commercial copper-based nanopesticides applied to plants and subsequently transferred to herbivorous caterpillars. In that plant–caterpillar system, copper transfer was associated with changes in host–microbiota interactions across trophic levels [12]. This finding moves the field beyond generic CuO NPs toxicity and into a realistic agricultural exposure scenario. It also suggests that copper-based nanopesticides may influence terrestrial food webs through both chemical transfer and microbiota-mediated host responses.

Food-web consequences may occur even when biomagnification is weak or absent. Limited transfer can still matter if copper-based nanopesticides alter prey quality, feeding behavior, growth, development, microbiota, or susceptibility to stress. Laboratory microcosm studies with multiple aquatic species have shown that Cu and CuO NPs can produce different uptake and toxicity profiles across algae, bacteria, crustaceans, and fish, indicating that community complexity can alter apparent nanoparticle risk [63]. Outdoor mesocosm experiments have shown that copper hydroxide nanopesticides can alter soil and sediment community diversity, with non-target wetland sediments appearing particularly sensitive [13]. Freshwater mesocosm work further shows that biotic and abiotic interactions can influence CuO NPs fate and zooplankton community dynamics over extended exposure periods [64]. More recently, copper nanopesticides were shown to reshape soil micro-food webs involving bacteria, fungi, protists, and nematodes, leading to trophic decoupling and altered nitrogen fluxes [14]. These findings shift the assessment endpoint from individual accumulation to food-web structure and ecosystem function.

Several uncertainties limit current interpretation. Many studies still measure total copper, which cannot distinguish dissolved copper, residual nanoparticles, transformed particles, and tissue-bound copper. Short-term food-chain experiments may also underestimate the influence of repeated application, aging, seasonal redox shifts, prey population dynamics, and predator feeding behavior. In addition, trophic transfer factors based on mass concentration may miss changes in particle number, particle size distribution, or particle transformation state. Particle-number-based trophic-transfer studies with other nanomaterials illustrate how mass-based metrics can overlook important features of nanoparticle movement through food chains [65]. For copper-based nanopesticides, this gap is especially relevant because dissolution and transformation can change both copper mass distribution and particle identity.

Studies with other engineered nanomaterials also show why food-chain design matters. Benthic transfer from clamworms to juvenile turbot has been demonstrated for TiO_2_ NPs, while surface functionalization altered ZnO NP uptake in *Daphnia magna* and transfer to zebrafish [66,67]. These analogs should not be treated as copper evidence. They are useful because they highlight design variables that remain underdeveloped in copper nanopesticide research, including prey ecology, particle surface chemistry, gut transformation, and predator digestive physiology.

Current evidence therefore supports a more nuanced interpretation of trophic transfer. Copper-based nanoforms can move through benthic, aquatic, and terrestrial food chains, but transfer efficiency and ecological consequences depend strongly on transformation state, prey ecology, gut processing, predator assimilation, and food-web structure. Benthic detrital pathways, biofilm–grazer interactions, plant–herbivore–natural enemy systems, and wetland micro-food webs remain underrepresented. Addressing these pathways is essential for determining when transformed copper becomes available for prey uptake, predator assimilation, food-web disruption, or ecosystem-function change.

## 7. Risk Assessment and Mitigation Strategies

Risk assessment of copper-based nanopesticides should begin with a clear problem formulation rather than a direct comparison between nanoforms and conventional copper salts. The relevant question is not only whether a nanopesticide is more or less toxic than dissolved copper. It is where the material moves after application, which copper species form during transport and aging, which organisms are exposed through water, sediment, food, or biofilms, and which ecological functions may be affected. This problem-formulation step is especially important for nano-enabled pesticides because exposure may involve the active ingredient, the nanoform, transformed products, or mixtures of these entities [68]. For copper-based nanopesticides, the benthic interface should be treated as a priority exposure compartment because runoff-derived particles can accumulate, transform, and become available to sediment-associated organisms and food webs.

A transformation-aware assessment should combine concentration, form, route, and time. Total copper remains necessary for mass balance and regulatory comparability, but it is insufficient for predicting bioavailability or food-web movement. A stronger assessment would include dissolved Cu, particle-specific measurements, porewater chemistry, sediment extractability, aging status, and speciation-sensitive tools. The aim is not to add analytical complexity for its own sake. The aim is to determine whether observed effects are driven by residual particles, dissolved copper, sulfur-associated phases, organic complexes, sediment-bound copper, gut-labile copper, or prey-assimilated copper. This distinction is central to copper-based nanopesticides because nanoformulations can change environmental fate without necessarily reducing environmental impact [69].

Ecological endpoints also need to move beyond standard single-species apical responses. Mortality, growth, and reproduction remain necessary, but they do not fully represent lower-trophic exposure or food-web risk. Feeding activity, burrowing, locomotion, avoidance behavior, gut microbiota, neurotoxicity, life-stage sensitivity, transgenerational effects, trophic transfer, and community structure are more informative when copper-based nanopesticides enter sedimentary or detrital pathways. Mesocosm and micro-food-web studies are particularly valuable because they can reveal effects on community diversity, zooplankton dynamics, trophic interactions, and nutrient fluxes that single-species assays may miss [13,14]. Long-term freshwater mesocosms further show that abiotic and biotic interactions can shape CuO NPs fate and ecological responses over time [64].

Risk assessment should also account for cumulative loading and temporal dynamics. Copper is an element and is not degraded like many organic pesticides. Repeated application may therefore shift risk from short-term crop protection to long-term accumulation in soils, sediments, and receiving waters. This issue is well recognized for conventional copper-based fungicides, where repeated use has been linked to elevated copper burdens in agricultural soils. For nano-enabled copper products, the same concern remains, but the relevant exposure forms may additionally include persistent particles, transformed particles, and nanoformulation-derived residues. A comprehensive environmental risk assessment is therefore needed before widespread agricultural application, especially when transformation, persistence, and chronic exposure remain uncertain [70].

Life-cycle risk assessment provides one way to connect material design with environmental consequences. Instead of evaluating only toxicity after release, a life-cycle approach can incorporate production, application, runoff, environmental transformation, exposure, effect characterization, and downstream ecological impact. Recent work has proposed a life-cycle risk assessment framework for nanopesticides in freshwater systems, allowing nanopesticide alternatives to be evaluated across more realistic environmental scenarios [71]. For copper-based nanopesticides, this approach is useful because risk may shift among compartments. A formulation that improves foliar efficacy may still increase benthic exposure if it enhances off-site transport, slows dissolution, or promotes long-term sediment retention.

Mitigation should start at the formulation and application stages. Safer-by-design copper nanopesticides should maintain crop-protection efficacy while reducing unnecessary copper loading, uncontrolled dissolution, long-term persistence, and off-site transport. This requires formulation assessment under realistic soil, runoff, and sediment conditions, not only in water or pathogen-inhibition assays. Recent discussions of nano-enabled pesticides emphasize that improved efficacy must be evaluated together with environmental release, persistence, transformation, and non-target exposure [1,69]. Application management can also reduce risk. Dose optimization, improved spray targeting, and avoidance of application before heavy rainfall are source-control measures that directly reduce the mass of Cu available for off-site transport. Their effects on nanopesticide-derived benthic exposure, however, still require field-scale validation.

Evidence for receiving-environment mitigation remains uneven. None of the measures reviewed here has yet been directly validated through a field-scale mass balance linking copper-based nanopesticide retention with reduced benthic exposure and ecological effects. Vegetated buffer strips are supported mainly by studies of conventional pesticides and sediment-associated runoff, where they reduce transport by slowing flow and trapping suspended material [72]. This provides an indirect mechanistic basis for particle-bound Cu, but not direct validation for copper-based nanopesticides.

Constructed wetlands and sediment traps may retain Cu-bearing particles, while biochar can reduce dissolved Cu or plant toxicity through sorption [73,74]. Nanobiochar has also been shown to alter the agronomic behavior of CuO-containing treatments [75]. These findings support potential retention or immobilization, but they do not demonstrate durable risk reduction for transformed nanopesticides. Buffer strips, wetlands, sediment traps, and biochar should therefore be classified as indirectly supported measures.

Retention can also displace exposure rather than eliminate it. Long-term operation of metal-removal wetlands has been associated with progressive Cu accumulation in surface sediments [74]. Such retention may reduce downstream water-column concentrations while increasing chronic exposure within the depositional zone. Similarly, lower porewater Cu does not necessarily reduce dietary exposure when sediment-ingesting organisms acquire particle-bound or aged Cu [28]. Permanent immobilization, absence of remobilization, and reduced food-web exposure should therefore be treated as hypotheses until they are tested under redox cycling, resuspension, and biological ingestion.

A defensible risk framework for copper-based nanopesticides should therefore link formulation design, agricultural use, environmental release, transformation, exposure, biological effect, and mitigation performance. The central benchmark should not be whether a nanoform is simply safer or more hazardous than a conventional copper product under one laboratory condition. The more relevant benchmark is whether improved agronomic efficiency can be achieved without increasing long-term exposure of benthic organisms, sediment communities, and food webs. This requires testing copper-based nanopesticides under realistic transformation scenarios, verifying mitigation performance in receiving environments, and interpreting ecological responses at organism, community, and food-web levels.

## 8. Conclusions and Future Perspectives

Copper-based nanopesticides may improve crop protection efficiency, but their environmental risk cannot be judged from pristine particles or total copper concentration alone. After application, copper nanoforms can move from crop surfaces to soils, drainage waters, wetlands, and sediments, where aggregation, dissolution, aging, sulfidation, organic complexation, and biological processing generate a continuum of copper species. These transformed species determine copper mobility, bioavailability, biological uptake, and food-web relevance.

The benthic interface is a critical control point in this process. It is not only a sink for runoff-derived particles but also a reactive zone where sediments, porewater, biofilms, roots, microbes, and benthic organisms jointly reshape copper speciation. Lower-trophic organisms are therefore central to risk interpretation because they integrate exposure from water, sediment, food, and biofilms. They may also transform, retain, excrete, or transfer copper species to higher trophic levels.

A key conclusion is that trophic transfer should not be equated with universal biomagnification. Copper-based nanoforms can move through sediment-based, aquatic, and terrestrial food chains, but transfer efficiency depends on copper speciation, aging, prey uptake, gut processing, egestion, predator assimilation, and food-web structure. A mass-based particulate-Cu TTF above one has been reported in one simulated aquatic chain [60], but most available studies demonstrate transfer or accumulation rather than consistently increasing Cu concentrations across trophic levels. Ecological consequences may also occur without strong biomagnification if copper nanopesticides alter prey quality, microbiota, community composition, trophic interactions, or nutrient cycling.

Future studies should move from simple exposure systems toward transformation-aware and food-web-relevant designs. Priority should be given to aged and sediment-associated materials, diet-borne exposure, lower-trophic organisms, isotope tracing, particle-specific measurements, and synchrotron-resolved speciation. Risk assessment should also be grounded in problem formulation, life-cycle thinking, mesocosm validation, runoff mitigation, and realistic receiving-environment scenarios. The next generation of studies should ask not only whether copper-based nanopesticides are more or less toxic than conventional copper products, but when, where, and in what chemical form copper becomes bioavailable, biologically processed, and ecologically consequential.

## Figures and Tables

**Table 1:** Major transformation processes of copper-based nanopesticides at the benthic interface.

Process	Main Drivers	Dominant Cu Species or Forms	Expected Effect on Mobility and Bioavailability	References
Aggregation and sedimentation	Ionic strength, electrolyte valence, pH, natural organic matter, clay minerals	Particle-bound Cu, heteroaggregates with minerals or organic matter	Reduces water-column mobility but increases sediment accumulation and benthic exposure	[19,20]
Dissolution	pH, dissolved organic matter, organic acids, biological ligands, coating, aging time	Cu^2+^, Cu–ligand complexes	Increases dissolved or labile Cu pools and may enhance acute bioavailability	[18,21,22]
Sulfidation	Reduced sulfur species, anoxic sediment, microbial sulfate reduction	Cu_x_S, CuS, sulfur-associated Cu	Generally reduces solubility and short-term bioavailability, but may be remobilized by oxidation, ingestion, or resuspension	[8]
Organic complexation	Humic substances, extracellular polymeric substances, thiols, reduced sulfur ligands, root exudates, and microbial metabolites	Cu–organic matter complexes, Cu–thiol complexes, ligand-stabilized Cu(I), colloidal Cu	Can reduce free Cu^2+^ while maintaining colloidal mobility or biological accessibility	[20,22,24]
Aging and sediment binding	Contact time, mineral surfaces, organic matter, redox gradients, sediment texture	Mineral-associated Cu, less labile sediment-bound Cu, transformed particles	Often decreases short-term bioavailability, but does not eliminate dietary exposure	[27,28]
Biological transformation and remobilization	Microbial activity, plant roots, gut fluids, burrowing, bioturbation, resuspension	Mixed Cu species, transformed particles, dissolved and complexed Cu	Can redistribute copper across sediment, porewater, organisms, and overlying water	[28,29]

**Table 2:** Evidence provided by synchrotron techniques and their principal limitations in copper speciation studies.

Technique	Evidence Provided	Principal Qualification	References
μ-XRF/SR-XRF	Elemental distribution and location of Cu-enriched regions	Spatial resolution is beamline- and matrix-dependent; elemental hotspots do not identify oxidation state, coordination, or intact nanoparticles.	[31,33,34,39]
XANES	Oxidation state and near-edge coordination environment	Phase assignment depends on suitable reference spectra and may be ambiguous when Cu environments overlap.	[8,9,30,31,32,35,36,37,38]
EXAFS	Neighboring atoms, bond distances, and local structural order	Requires a stronger signal and structural modeling; it does not independently establish particle morphology or origin.	[8,24,30,31,32]

**Table 3:** Evidentiary interpretation of representative trophic-transfer studies involving copper-based nanoforms.

Exposure Chain	Measurement or Evidence	Supported Interpretation	References
Sediment–*Tubifex*–fish	^65^Cu tracing through sediment, prey, and predator	Worm accumulation and prey-to-predator transfer; biomagnification not independently demonstrated.	[10]
Worm-derived food–fish	Fish Cu burden and fecal Cu after controlled dietary exposure	Dietary transfer with substantial egestion of particulate Cu.	[59]
Microalga–daphnid–mysid	Particle mass and number measured by sp-ICP-MS; mass-based TTF > 1 from alga to mysid	Biomagnification under the study-specific particulate-Cu mass metric.	[60]
*Artemia*–fish larvae	Whole-body Cu during uptake and elimination	Prey-mediated transfer and whole-body accumulation; tissue-specific assimilation unresolved.	[11]
Contaminated prey or diet–fish	Tissue Cu after dietary and waterborne exposure	Route-specific accumulation; not a direct trophic-magnification test.	[61,62]
Plant–caterpillar	Time-resolved Cu transfer after nanopesticide application	Terrestrial trophic transfer; biomagnification not established.	[12]

Note: TTF definitions and normalization differed among studies. A consumer-to-prey ratio above one should be interpreted as biomagnification only when comparable tissues, depuration procedures, and wet- or dry-weight bases are used. No common TTF was recalculated in this review.

## Data Availability

No new data were created or analyzed in this study. Data sharing is not applicable to this article.
